# Timing of renal replacement therapy in acute kidney injury after liver transplantation: the clinical value of the NMF standards

**DOI:** 10.3389/fmed.2026.1737583

**Published:** 2026-06-18

**Authors:** Gong-jie Ye, Zhou-zhou Dong

**Affiliations:** Department of Intensive Care Unit, The Affiliated Lihuili Hospital of Ningbo University, Ningbo, Zhejiang, China

**Keywords:** acute kidney injury, clinical value, liver transplantation, renal replacement therapy, timing selection

## Abstract

**Objective:**

To evaluate the utility of the NMF standards—comprising intraoperative norepinephrine dosage, preoperative MELD-Na score, and total intraoperative fluid intake—in determining the optimal timing for initiating renal replacement therapy (RRT) in patients with acute kidney injury (AKI) following liver transplantation.

**Methods:**

The NMF standards were developed and validated in two studies: (1) a retrospective cohort study (2018–2023, 189 patients with KDIGO stage 2 AKI) stratified by NMF status and RRT timing into four groups (A, B, C, D); and (2) a historically controlled cohort study (2024–2025, 76 NMF-guided patients vs. 189 historical controls managed by clinical judgment). The primary outcomes included renal function indicators, hospital stay, CKD incidence, and long-term RRT dependence.

**Results:**

The NMF standards have been validated as a robust clinical tool: intraoperative norepinephrine ≥6.5 μg·kg^−1^·h^−1^, preoperative MELD-Na ≥15.5, and total fluid intake ≥6,600 ml. In the retrospective cohort, patients meeting NMF standards who received timely RRT exhibited significantly better renal function, lower blood ammonia, shorter hospital stay, and lower rates of CKD and long-term RRT dependence than those with delayed or no RRT. For patients not meeting the NMF standards, early RRT did not show significant long-term benefits in this small subgroup, but was associated with an increased risk of catheter-related bloodstream infection. In the historically controlled cohort study, NMF-guided RRT significantly improved 7-day creatinine (64.5 vs. 72.2 μmol/L), shortened hospital stay (24.0 vs. 27.5 days), and reduced CKD (14.5% vs. 24.5%) and long-term RRT dependence (7.9% vs. 18.5%) compared with clinically guided RRT.

**Conclusions:**

The NMF standards, integrated with a user-friendly WeChat mini-program, provide a validated and practical framework for optimizing RRT timing in AKI post-liver transplantation. This tool enhances clinical decision-making, reduces complications, and improves long-term renal outcomes.

## 1 Introduction

Liver transplantation is recognized the optimal treatment for end-stage liver diseases arising from various prevalent causes, including hepatitis B cirrhosis, alcoholic, drug-induced, and autoimmune liver diseases (1). AKI represents a frequent complication following liver transplantation (2, 3). Preventing AKI post-transplantation poses substantial challenges. Some researchers focus on mitigating the onset of hepatorenal syndrome by monitoring liver and kidney function preoperatively and by avoiding or cautiously administering nephrotoxic drugs, such as lactulose, contrast agents, non-steroidal anti-inflammatory drugs, and cyclooxygenase-2 inhibitors. However, these measures have failed to achieve ideal outcomes. The development of AKI significantly extends the duration of mechanical ventilation, ICU stay, and overall hospitalization for recipients, while also increasing the incidence and overall mortality of CKD (4, 5). RRT remains the most commonly employed and effective treatment for AKI in post-liver transplantation patients, with no other definitive therapies currently available (6).

The main challenge in managing AKI in patients following liver transplantation is to maintain kidney function while optimizing fluid balance, which involves determining the optimal timing for the initiation of RRT. Initiating RRT early can effectively alleviate the body's excessive fluid load, correct metabolic acidosis, enhance oxygenation, mitigate complications such as metabolic encephalopathy and gastrointestinal bleeding, and reduce the duration of mechanical ventilation and ICU stay (6, 7). Conversely, other scholars argue that premature initiation of RRT may elevate the risk of infections in transplant recipients, particularly CRBSI (8), and that mortality rates do not significantly differ from those associated with conservative management or delayed RRT (9, 10), early RRT may only contribute to increased patient discomfort and health-care costs.

Currently, the literature on the relationship between the timing of RRT and prognosis in patients with AKI following liver transplantation is predominantly derived from observational studies (11). The potential for conservative management or delayed RRT to allow for spontaneous renal recovery has not been thoroughly investigated. Determining whether the residual renal function in AKI patients is adequate to sustain electrolyte and acid-base homeostasis, as well as the clearance of metabolic waste, and identifying the optimal timing for initiating RRT, largely depends on clinical expertise and remains a topic of considerable debate. Our team previously performed a retrospective analysis of risk factors associated with postoperative AKI and the initiation of RRT in liver transplant recipients during the early postoperative period. Our findings indicated that intraoperative NE dosage (equivalent conversion of other vasoactive drugs to norepinephrine dosage), preoperative MELD-Na score, and total intraoperative fluid intake are independent risk factors for postoperative AKI and RRT in this patient population (12). These results are consistent with findings from multiple studies conducted both domestically and internationally (13, 14).

Our findings indicate that relying solely on the KDIGO staging criteria, which are based on creatinine levels and urine output, as the primary determinant for initiating RRT may lead to the misclassification of a substantial number of patients. Such misclassification could potentially hinder improvements in renal function recovery and survival rates (12). Moreover, there is ongoing debate concerning the optimal timing for RRT initiation during the second phase of AKI. Based on our previous work, this study integrated KDIGO staging with three independent risk factors to develop the “NMF standards” and evaluate its effectiveness in determining the optimal timing for RRT initiation in patients experiencing AKI following liver transplantation.

## 2 Materials and methods

### 2.1 Development of the NMF standards

The core indicators and corresponding threshold values of the NMF standards were derived from our previous preliminary research (12). Briefly, multivariate logistic regression was used to screen independent risk factors requiring RRT after liver transplantation, and ROC curve analysis combined with the Youden index was applied to calculate the optimal cut-off values: intraoperative norepinephrine ≥6.5 μg·kg^−1^·h^−1^, preoperative MELD-Na ≥ 15.5, and intraoperative total fluid intake ≥6,600 ml. The MELD-Na score calculated as the official UNOS/OPTN version: The MELD-Na score is an integrated measure combining the Model for End-Stage Liver Disease (MELD) with serum sodium levels. It is calculated as MELD + 1.32 × (137 – Na^+^) – 0.033 × MELD × (137 – Na^+^). When the serum sodium (Na^+^) concentration exceeds 137 mmol/L, it is capped at 137 mmol/L for calculation purposes. For Na^+^ levels below 125 mmol/L, the calculation uses 125 mmol/L, while actual values are used for concentrations between 125 and 137 mmol/L.

In terms of judgment logic, intraoperative norepinephrine dosage was set as the primary indicator reflecting real-time hemodynamic disturbance, whereas preoperative MELD-Na and intraoperative fluid overload were regarded as secondary risk indicators. The NMF standards were defined as positive when patients met one primary indicator or two secondary indicators concurrently, which balances clinical practicability and risk screening efficiency.

### 2.2 Verify the feasibility of NMF standards

#### 2.2.1 Study type and design

This study was a combined retrospective cohort study and historically controlled cohort study conducted in a single center. Retrospective cohort: January 2018–December 2023. Historically controlled cohort: January 2024–September 2025.

#### 2.2.2 Sample size calculation and power analysis

The sample size was estimated based on the primary endpoint postoperative 7-day serum creatinine and incidence of CKD. A power analysis was performed with a type I error α = 0.05 (two-sided) and statistical power = 80%. According to previous studies and our preliminary data, a clinically significant difference in creatinine of ≥15 μmol/L and a 10% difference in CKD incidence were considered meaningful. The required minimum sample size was 178 patients in the retrospective part and 72 patients in the prospective part. Finally, 189 retrospective patients and 76 historically controlled patients were enrolled to ensure sufficient statistical efficiency and avoid loss to follow-up.

#### 2.2.3 Data collection tools and collected patient characteristics

Data collection tools: Clinical data were retrospectively or prospectively collected via the electronic medical record system of ICU and transplant center. All data were extracted by two trained independent researchers using a standardized case report form (CRF). Discrepancies were resolved by a third senior investigator.

Patient characteristics collected included the following items: (1) Basic information, which includes the patient's age, gender, body mass index (BMI), etiology, SOFA score, APACHE II score, timing of transplant surgery, surgical method (whole liver transplantation or split liver transplantation), and the presence of hepatic coma. (2) Clinical indicators, which encompass creatinine levels, MELD-Na scores, and blood ammonia assessed preoperatively, postoperatively, and on the 1st, 3rd, and 7th days following transplantation surgery. (3) Prognostic indicators, which consist of cumulative mechanical ventilation duration, cumulative ICU stay, total hospital stay, 28-day mortality rate, 90-day mortality rate, one-year mortality rate, incidence of tracheotomy, CRBSI, CKD, requirement for long-term RRT, RRT-related adverse events, and presence of liver dysfunction.

#### 2.2.4 Outcome definitions

Long-term RRT dependence was defined as the need for renal replacement therapy (RRT) for ≥90 consecutive days after liver transplantation. CKD was defined according to the 2012 KDIGO criteria (15): estimated glomerular filtration rate (eGFR) < 60 ml·min^−1^·1.73 m^−2^ persisting for ≥90 days after transplantation. All patients were followed up at 1, 3, and 6 months after discharge.

Timely RRT was defined as RRT initiation within 6 h of meeting the NMF standards. This cutoff was chosen because early intervention within 6 h effectively prevents progressive renal injury and systemic inflammation in critically ill patients with AKI, supported by previous clinical evidence (10, 16).

#### 2.2.5 Inclusion and exclusion criteria

Inclusion criteria were as follows: (1) Diagnosis of AKI post-LTx as per the 2012 KDIGO guidelines (15), specifically KDIGO stage 2, characterized by a creatinine increase to 2.0–2.9 times the baseline level or a urine output of 0.5 ml/kg/h sustained for at least 12 h; (2) Recipient age of 18 years or older; (3) All donors must be brain-dead, as defined by the 2013 “Criteria and Technical Regulations for Brain Death Determination (Adult Quality Control Edition).” Exclusion criteria included: (1) Patients with a history of CKD necessitating long-term hemodialysis or peritoneal dialysis; (2) Recipients of combined liver-kidney transplantation; (3) Individuals unable to undergo RRT due to specific contraindications.

#### 2.2.6 The retrospective cohort study

Between January 2018 and December 2023, patients who developed AKI following LTx and were admitted to the ICU were assessed for inclusion based on specific criteria. Eligible patients were subsequently divided into four distinct groups (A, B, C, D) for a retrospective cohort study. Groups A and B comprised one cohort, focusing on evaluating whether patients who satisfied the NMF standards and underwent RRT demonstrated particular benefits. Conversely, Groups C and D constituted another cohort, aimed at exploring potential disadvantages in patients who did not meet the NMF standards but still received RRT. The overarching objective of this study was to thoroughly assess the utility of the “NMF standards.” Group A included patients who met the NMF standards and received RRT promptly, within less than 6 h post-admission. Group B consisted of patients who met the NMF standards but either did not receive RRT or received it with a delay of 6 h or more. Group C encompassed patients who did not meet the NMF standards but received RRT immediately, within less than 6 h after admission. Lastly, Group D comprised patients who did not meet the NMF standards and either did not receive RRT or received it with a delay of 6 h or more.

#### 2.2.7 The historically controlled cohort study

The NMF Group: From January 2024 to September 2025, ICU patients with AKI classified as KDIGO stage 2 after LTx will be selected and assessed using NMF standards to determine if RRT should be initiated within 6 h of admission. Control Group: Between January 2018 and December 2023, similar patients were evaluated based on the attending physician's clinical judgment for RRT initiation within 6 h. A historically controlled cohort study will compare these groups to evaluate the feasibility and efficacy of the NMF standards.

#### 2.2.8 Ethical informed consent form

The Institutional Ethical Committee approved the study protocol (Medical ethical committee of Ningbo Medical Center Lihuili Hospital, Ethics Number: KY2022SL262-01). Trial Registration: China National Health Security Information Platform Medical Research Registration and Filing Information System, Record number: MR-33-25-042230. Informed consent was obtained from the patient's guardian. All procedures were performed in accordance with relevant approved guidelines and regulations.

#### 2.2.9 Statistical analysis

All statistical analyses were performed using SPSS 26.0 and Python 3.9. Data were tested for normality and non-Gaussian distribution using the *Kolmogorov-Smirnov* test. Normally distributed data were presented as *mean* ± *SD*, while non-normally distributed data are expressed as median [*25–75%, interquartile range (IQR)*]. Both parametric and non-parametric methods were employed in the analysis. Inter-group comparisons were performed utilizing either the *t-test* or analysis of variance (*ANOVA*). Categorical data were represented as percentages, and comparisons were executed using a *chi-square* (χ^2^) test within a contingency table framework. For metric and ordinal data that did not satisfy the assumptions required for the *t-test*, χ^2^ *test*, or *ANOVA*, a non-parametric rank sum test was employed. A significance level of α = 0.05 was adopted, with differences were considered statistically significant when *P* < 0.05.

Multivariate adjustment: Multivariate linear regression was used for continuous outcomes, and binary logistic regression was used for categorical outcomes. Covariates with *P* < 0.2 in univariate analysis were included in the adjusted models. Propensity score matching (PSM) was performed as 1:1 nearest-neighbor matching with a caliper of 0.05 was performed to balance baseline characteristics. The propensity score was estimated using a logistic regression models. Outcomes were re-analyzed in the matched cohort. A two-sided *P* < 0.05 was considered statistically significant.

## 3 Results

### 3.1 Develop NMF standards and a WeChat mini program

The relevant tasks in the preliminary phase have been completed. In accordance with NMF standards, as outlined in Table 1, RRT was initiated when a patient satisfied either one primary criterion or two secondary criteria.

**Table 1:** NMF standards for AKI patients after liver transplantation.

Parameters	Articles
Primary criterion	The dosage of NE during the operation ≥6.5μg·kg^−1^·h^−1^
Secondary criteria	MELD-Na score before surgery ≥15.5
	The total intraoperative intake volume ≥6600 ml

NE, norepinephrine. The MELD-Na score is an integrated measure combining the model for end-stage liver disease (MELD) with serum sodium levels. It is calculated as MELD + 1.32 × (137 – Na^+^) – 0.033 × MELD × (137 – Na^+^). When the serum sodium (Na^+^) concentration exceeds 137 mmol/L, it is capped at 137 mmol/L for calculation purposes. For Na^+^ levels below 125 mmol/L, the calculation uses 125 mmol/L, while actual values are used for concentrations between 125 and 137 mmol/L. The MELD score itself is computed using the formula: 3.8 × ln(TBil) + 11.2 × ln(INR) + 9.6 × ln(Cr) + 6.4 × Cause, where TBil represents total bilirubin in mg/dl, INR denotes the international normalized ratio, Cr indicates creatinine in mg/dl, and Cause is a binary variable (0 for cholestasis or alcohol-related liver disease, 1 for other causes).

**Figure 1:**
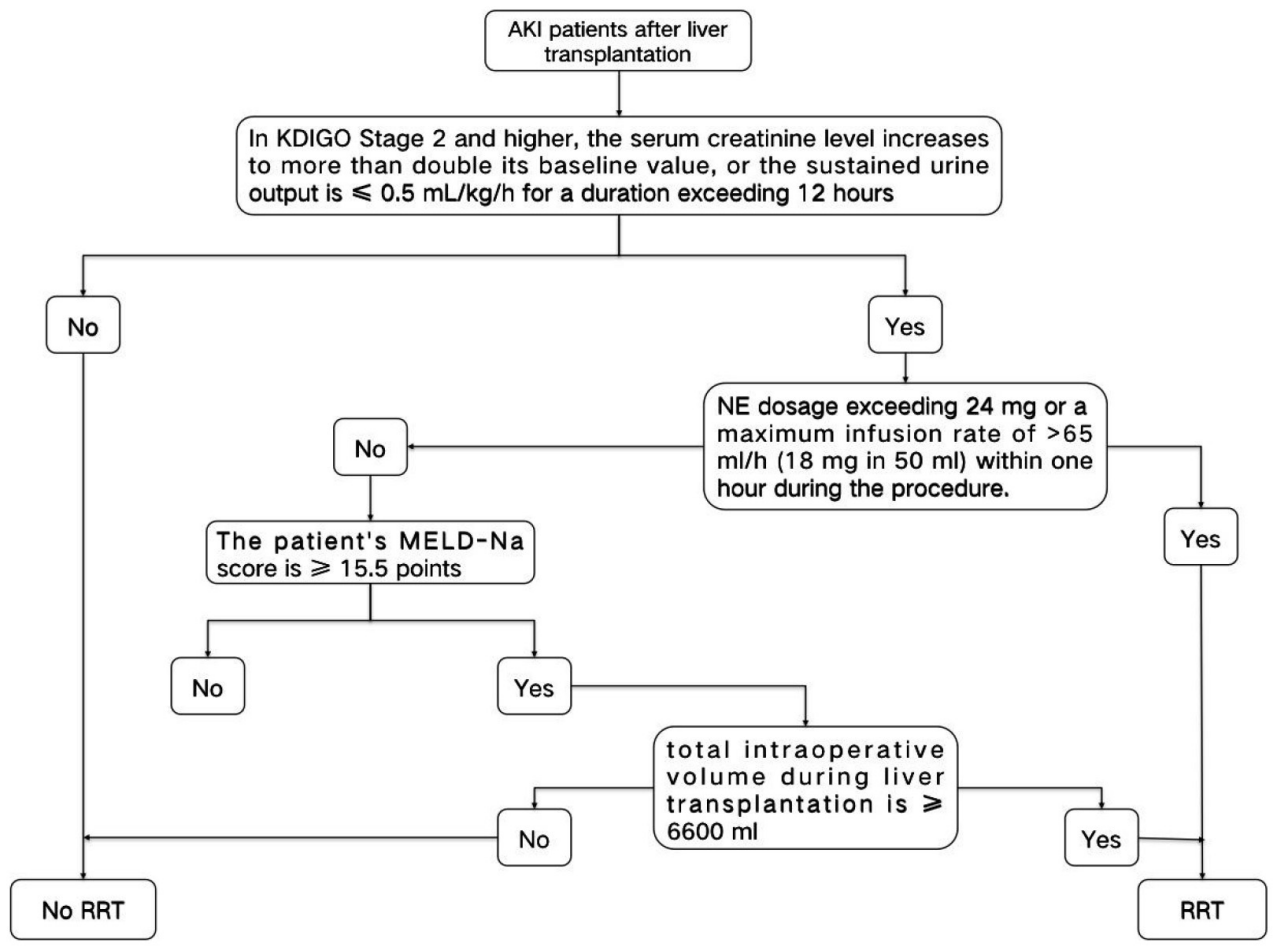
Flow chart of the NMF standards.

We developed a clinical decision-support tool based on the NMF standards to simplify risk stratification and RRT timing decisions. This tool enables automatic evaluation by inputting the three key parameters and provides a clear recommendation for RRT initiation. The software copyright of NMF Standards Risk Assessment Software V1.0 (registration number: 2025SR0409667) has been approved. The structure and workflow of the tool are shown in Figure 1.

### 3.2 Verify the feasibility of NMF standards

#### 3.2.1 The retrospective cohort study

##### 3.2.1.1 Basic information

**Figure 2:**
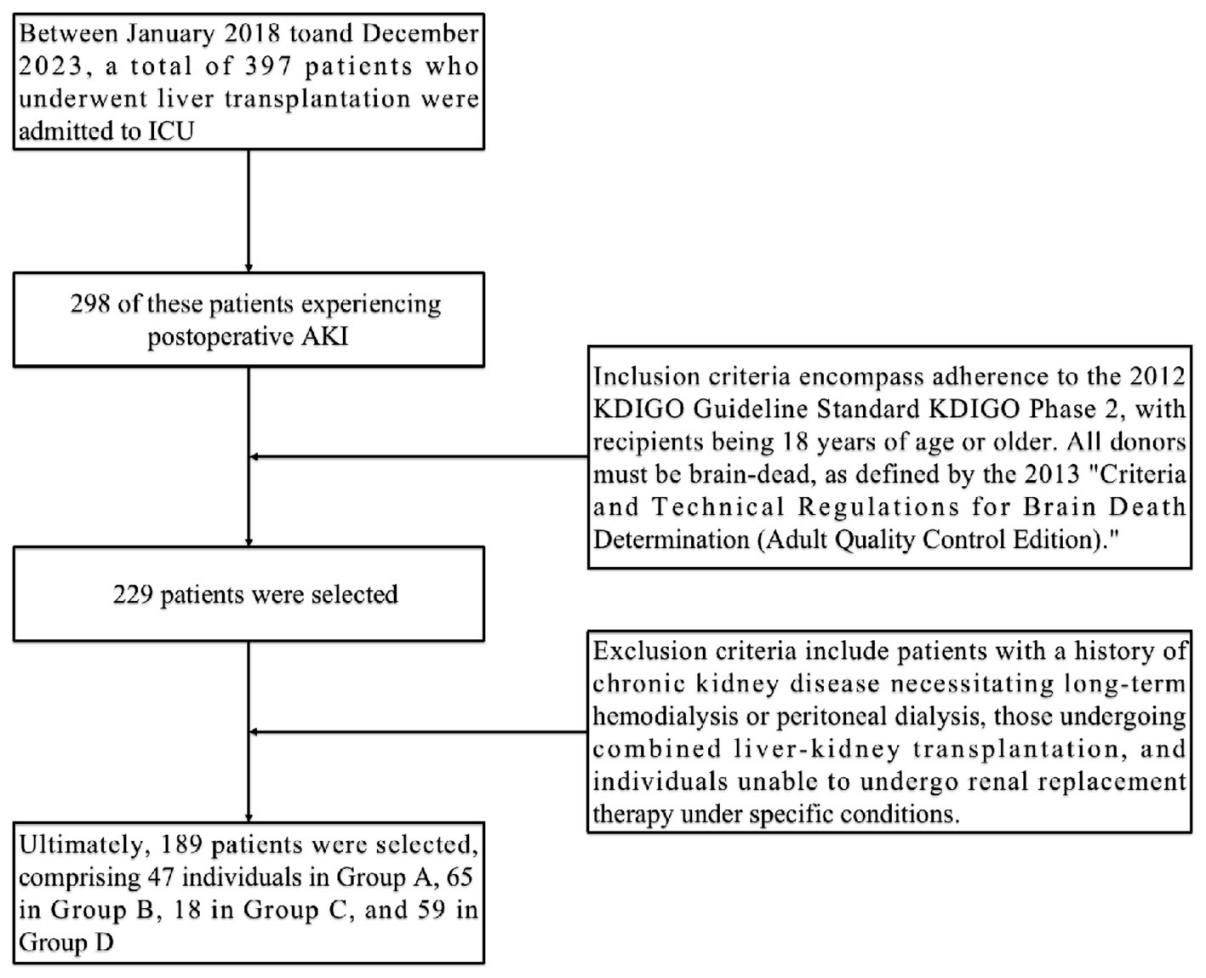
NMF standards: flowchart for inclusion and exclusion of research subjects.

Between January 2018 and December 2023, a total of 397 liver transplant recipients were admitted to the ICU at our hospital. Among these patients, 298 developed postoperative AKI, and 229 were classified as having KDIGO stage 2. Based on the established inclusion and exclusion criteria, 189 patients were ultimately selected for the study. These individuals were categorized into four groups: 47 patients in Group A, 65 in Group B, 18 in Group C, and 59 in Group D. Further details can be found in Figure 2.

No significant between-group differences were observed in age, gender, body mass index (BMI), etiology, SOFA score, APACHE II score, duration of surgery, surgical method (whole liver transplantation vs. split liver transplantation), or the presence of hepatic coma between groups A and B, as well as between groups C and D (*P* > 0.05), as presented in Table 2.

**Table 2:** Basic information between group A and group B, group C and group D (*x̄* ± *s*) or *M* (*Q*_25_, *Q*_75_).

Parameters	Group A (*n* = 47)	Group B (*n* = 65)	*P*	Group C (*n* = 18)	Group D (*n* = 59)	*P*
Age (year)	52.00 ± 9.43	52.23 ± 7.78	0.89	53.94 ± 7.54	53.02 ± 10.96	0.74
Male [*n* (%)]	38 (80.85)	58 (89.23)	0.21	15 (83.33)	53 (89.83)	0.43
BMI (kg/m^2^)	22.66 ± 3.56	22.56 ± 2.71	0.87	22.49 ± 3.16	21.74 ± 2.61	0.32
Etiology [*n* (%)]						
Virus	35 (74.47)	48 (73.85)	0.94	13 (72.22)	44 (74.58)	0.99
Alcoholic	8 (17.02)	11 (16.92)	0.99	3 (16.67)	9 (15.25)	1.00
Drugs or toxins	2 (4.26)	4 (6.15)	0.99	1 (5.56)	4 (6.78)	0.99
Others	2 (4.26)	2 (3.08)	0.99	1 (5.56)	3 (5.08)	1.00
SOFA (score)	5.00 (3.00, 6.00)	4.00 (2.50, 6.00)	0.27	3.56 ± 1.15	4.14 ± 1.96	0.24
APACHE II (score)	11.02 ± 4.36	10.46 ± 5.06	0.54	10.28 ± 2.11	10.83 ± 3.93	0.57
Duration of transplant surgery (min)	524.74 ± 106.23	537.86 ± 107.16	0.52	538.9 ± 96.2	531.4 ± 107.9	0.79
Whole liver transplantation [*n* (%)]	27 (57.45)	43 (66.15)	0.35	13 (72.22)	37 (62.71)	0.46
Preoperative hepatic coma [*n* (%)]	6 (12.77)	8 (12.31)	0.94	2 (11.11)	10 (16.95)	0.72

BMI, body mass index; SOFA, sequential organ failure score; APACHE-II, acute physiology and chronic health II score.

##### 3.2.1.2 Clinical indicators

In Groups A and B, no significant differences were observed in creatinine levels, MELD-Na scores, or blood ammonia concentrations before and after the operation, as well as in MELD-Na scores on the first and third postoperative days (*P* > 0.05). However, significant differences were found in creatinine levels and blood ammonia concentrations on the first and third postoperative days, and in creatinine levels, MELD-Na scores, and blood ammonia concentrations on the seventh postoperative day (*P* < 0.05). In Groups C and D, no significant differences were observed in creatinine levels, MELD-Na scores, or blood ammonia concentrations before the operation, immediately after the operation, or on the seventh postoperative day, as well as in MELD-Na scores on the first and third postoperative days (*P* > 0.05). Nevertheless, significant differences were detected in creatinine levels on the first postoperative day and in creatinine levels and blood ammonia concentrations on the third postoperative day (*P* < 0.05). For further details, refer to Table 3.

**Table 3:** Clinical indicators between group A and group B, group C and group D (*x̄* ± *s*) or *M* (*Q*_25_, *Q*_75_).

Parameters	Group A (*n* = 47)	Group B (*n* = 65)	*P*	Group C (*n* = 18)	Group D (*n* = 59)	*P*
Preoperative						
Creatinine (μmol/L)	68.10 (56.00, 85.70)	68.10 (54.05, 85.55)	0.91	65.78 ± 22.47	70.29 ± 31.97	0.58
MELD-Na (score)	7.95 (4.89, 16.56)	10.18 (4.99, 16.37)	0.64	12.52 (8.12, 13.61)	10.18 (5.08, 15.98)	0.51
Blood ammonia (μmol/L)	39.00 (29.00, 47.00)	36.00 (28.00, 44.00)	0.46	39.00 (26.00, 58.00)	37.00 (30.00, 44.00)	0.61
Postoperative						
Creatinine (μmol/L)	175.86 ± 63.70	169.76 ± 67.91	0.63	88.26 ± 21.64	81.23 ± 32.15	0.38
MELD-Na (score)	12.68 (10.35, 15.96)	12.83 (9.89, 18.19)	0.87	12.69 ± 3.37	12.62 ± 4.15	0.95
Blood ammonia (μmol/L)	40.00 (31.00, 47.00)	38.00 (26.00, 50.50)	0.43	36.00 (22.75, 42.75)	37.00 (27.00, 50.00)	0.59
Postoperative days 1						
Creatinine (μmol/L)	111.26 ± 41.52	143.29 ± 55.99	< 0.01	61.65 (54.60, 69.28)	92.10 (61.20, 119.40)	< 0.01
MELD-Na (score)	13.96 (9.93, 17.29)	14.37 (9.89, 16.90)	0.91	11.44 ± 4.35	12.37 ± 3.33	0.34
Blood ammonia (μmol/L)	25.00 (20.00, 35.00)	38.00 (26.00, 50.50)	< 0.01	31.00 (19.75, 48.75)	40.00 (32.00, 47.00)	0.06
Postoperative days 3						
Creatinine (μmol/L)	79.40 (68.90, 93.40)	100.10 (71.80, 153.10)	0.01	63.10 (57.85, 78.65)	79.20 (59.40, 109.70)	0.03
MELD-Na (score)	12.62 (8.02, 17.92)	13.85 (9.20, 20.48)	0.26	12.78 ± 3.31	12.63 ± 5.18	0.91
Blood ammonia (μmol/L)	20.00 (15.00, 23.00)	43.00 (33.00, 55.00)	< 0.01	19.50 (14.00, 22.75)	43.00 (33.00, 55.00)	< 0.01
Postoperative days 7						
Creatinine (μmol/L)	68.60 (62.30, 73.50)	91.20 (71.80, 160.05)	< 0.01	69.25 (55.08, 89.18)	69.10 (54.00, 86.40)	0.62
MELD-Na (score)	8.60 (6.97, 16.21)	14.58 (8.59, 21.59)	0.01	7.58 (6.03, 15.24)	9.87 (7.13, 16.61)	0.22
Blood ammonia (μmol/L)	12.00 (10.00, 16.00)	37.00 (21.00, 47.00)	< 0.01	22.83 ± 7.29	25.24 ± 8.77	0.30

MELD-Na, Model for end-stage liver disease combined with serum sodium.

In Groups A and B, no significant differences were observed in the cumulative duration of mechanical ventilation, cumulative length of ICU stay, 28-day mortality, 90-day mortality, one-year mortality, incidence of CRBSI, or incidence of abnormal liver function (*P* > 0.05). However, significant differences were observed in the total length of hospital stay, incidence of CKD, and requirement for long-term RRT (*P* < 0.05). Conversely, in Groups C and D, no significant differences were identified in cumulative mechanical ventilation time, cumulative ICU length of stay, total hospital length of stay, 28-day mortality, 90-day mortality, one-year mortality, incidence of CKD, requirement for long-term RRT, or incidence of abnormal liver function (*P* > 0.05). A statistically significant difference was noted between patients with and without CRBSI (*P* < 0.05). Further details are provided in Table 4.

**Table 4:** Prognostic indicators between group A and group B, group C and group D (*x̄* ± *s*) or *M* (*Q*_25_, *Q*_75_).

Parameters	Group A (*n* = 47)	Group B (*n* = 65)	*P*	Group C (*n* = 18)	Group D (*n* = 59)	*P*
Cumulative mechanical ventilation duration (*d*)	0.63 (0.54, 0.75)	0.64 (0.51, 0.85)	0.67	0.58 (0.49, 0.90)	0.63 (0.48, 0.79)	0.70
Cumulative ICU stay (*d*)	2.48 (1.85, 3.71)	1.96 (1.67, 2.90)	0.34	2.73 (1.90, 3.72)	1.96 (1.71, 2.88)	0.11
Total hospital stay (*d*)	27.55 ± 9.13	35.35 ± 15.27	< 0.01	30.83 ± 13.41	28.80 ± 13.01	0.57
Tracheotomy [*n* (%)]	3 (6.38)	8 (12.31)	0.35	1 (5.56)	5 (8.47)	1.00
Twenty-eight-day mortality rate (%)	4 (8.51)	8 (12.31)	0.48	0 (0.00)	3 (5.08)	0.99
Ninety-day mortality rate (%)	5 (10.64)	11 (16.92)	0.31	0 (0.00)	3 (5.08)	0.99
One-year mortality rate (%)	8 (17.02)	13 (20.00)	0.63	1 (5.56)	4 (6.78)	1.00
CKD [*n* (%)]	10 (21.28)	24 (36.92)	0.03	4 (22.22)	14 (23.73)	1.00
CRBSI [*n* (%)]	14 (29.79)	10 (15.38)	0.08	6 (33.33)	6 (10.17)	0.03
Requirement for long-term RRT [*n* (%)]	6 (12.77)	19 (29.23)	0.04	3 (16.67)	10 (16.95)	1.00
Presence of liver dysfunction [*n* (%)]	26 (55.32)	38 (58.46)	0.70	8 (44.44)	35 (59.32)	0.78

ICU, intensive care unit; CKD, chronic kidney disease; CRBSI, catheter-related bloodstream infection; RRT, renal replacement therapy.

#### 3.2.2 The historically controlled cohort study

##### 3.2.2.1 Basic information

From January 2024 to September 2025, a cohort of patients admitted to the ICU of our hospital, who met the criteria for AKI following liver transplantation and were classified as KDIGO stage 2, underwent screening based on the NMF standards to assess the necessity for immediate RRT within 6 h of admission. A total of 76 patients were ultimately enrolled in the NMF group. The control group comprised 189 patients admitted to the ICU from January 2018 and December 2023, who also met the criteria for AKI post-liver transplantation and were classified as KDIGO stage 2. In the control group, the decision to initiate RRT within 6 h of admission was based on the attending physician's clinical judgment. Comparison between the NMF and control group revealed statistically significant differences (*P* > 0.05) in variables such as patient age, gender, BMI, etiology, SOFA score, APACHE II score, duration of transplant surgery, the occurrence of complete liver transplantation, and the presence of hepatic coma, as detailed in Table 5.

**Table 5:** Basic information between the NMF and control group (*x̄* ± *s*) or *M* (*Q*_25_, *Q*_75_).

Parameters	The NMF group (*n* = 76)	Control group (*n* = 189)	*t*/*U*/χ^2^	*P*
Age (year)	54.34 ± 9.35	52.82 ± 8.95	1.23	0.22
Male [*n* (%)]	62 (81.58)	158 (83.60)	0.16	0.69
BMI (kg/m^2^)	23.01 ± 3.28	22.36 ± 2.95	1.57	0.12
Etiology [*n* (%)]				
Virus	56 (73.68)	130 (68.78)	0.62	0.43
Alcoholic	11 (14.47)	30 (15.87)	0.08	0.78
Drugs or toxins	5 (6.58)	16 (8.47)	0.26	0.61
Others	4 (5.26)	13 (6.88)	0.24	0.79
SOFA (score)	4.00 (3.00, 6.00)	4.00 (3.00, 6.00)	0.25	0.80
APACHE II (score)	9.50 (7.00, 14.75)	10.00 (7.00, 13.00)	0.30	0.77
Duration of transplant surgery (min)	526.95 ± 101.19	530.63 ± 106.03	0.26	0.80
Whole liver transplantation [*n* (%)]	49 (64.47)	115 (60.85)	0.30	0.58
Preoperative hepatic coma [*n* (%)]	12 (15.79)	25 (13.23)	0.30	0.59

BMI, body mass index; SOFA, sequential organ failure score; APACHE-II, acute physiology and chronic health II score.

##### 3.2.2.2 Clinical indicators

No statistically significant differences were observed in creatinine levels, MELD-Na scores, and blood ammonia levels between the NMF group and control group in preoperative, postoperative, and on postoperative days 3, as well as in MELD-Na scores and blood ammonia levels on postoperative days 7 (*P* > 0.05). Conversely, statistically significant differences (*P* < 0.05) were identified between the two group in terms of creatinine levels, MELD-Na scores, and blood ammonia levels on postoperative days 1, as well as in creatinine levels on postoperative days 7, as detailed in Table 6.

**Table 6:** Clinical indicators between the NMF and control group (*x̄* ± *s*) or *M* (*Q*_25_, *Q*_75_).

Parameters	The NMF group (*n* = 76)	Control group (*n* = 189)	*t/U*	*P*
Preoperative				
Creatinine (μmol/L)	67.55 (53.93, 86.60)	66.90 (54.00, 83.20)	0.36	0.72
MELD-Na (score)	11.36 ± 4.48	11.62 ± 4.57	0.41	0.68
Blood ammonia (μmol/L)	36.30 ± 10.78	38.88 ± 11.44	1.68	0.10
Postoperative				
Creatinine (μmol/L)	146.65 (91.55, 165.53)	124.35 (79.48, 184.50)	0.17	0.87
MELD-Na (score)	13.93 ± 4.26	13.30 ± 4.03	1.12	0.26
Blood ammonia (μmol/L)	38.09 ± 9.37	38.57 ± 11.25	0.32	0.75
Postoperative days 1				
Creatinine (μmol/L)	85.85 (62.10, 128.60)	109.00 (72.40, 152.83)	2.22	0.03
MELD-Na (score)	14.94 ± 5.27	13.64 ± 4.29	2.07	0.04
Blood ammonia (μmol/L)	27.49 ± 13.22	37.53 ± 17.36	4.52	< 0.01
Postoperative days 3				
Creatinine (μmol/L)	79.45 (59.33, 109.63)	81.85 (63.65, 117.58)	0.87	0.39
MELD-Na (score)	12.91 ± 5.45	13.38 ± 5.00	0.67	0.51
Blood ammonia (μmol/L)	32.00 (23.00, 43.00)	34.00 (20.00, 45.00)	0.73	0.47
Postoperative days 7				
Creatinine (μmol/L)	64.45 (50.65, 86.38)	72.20 (61.78, 101.43)	3.35	< 0.01
MELD-Na (score)	12.08 ± 4.53	12.94 ± 5.77	1.16	0.25
Blood ammonia (μmol/L)	19.50 (15.00, 32.00)	22.00 (15.00, 37.25)	1.76	0.08

MELD-Na, model for end-stage liver disease combined with serum sodium.

##### 3.2.2.3 Prognostic indicators

A comparative analysis between the NMF and control group showed no statistically significant differences in cumulative duration of mechanical ventilation, cumulative ICU length of stay, incidence of tracheotomy, 28-day mortality, 90-day mortality rate, and CRBSI incidence (*P* > 0.05). Similarly, no significant between-group differences were observed in the overall incidence of RRT-related adverse events (including hemodynamic instability, circuit clotting, catheter-related complications, and bleeding events) or the prevalence of liver dysfunction (*P* > 0.05). Conversely, statistically significant differences were identified in total hospital stay, incidence of CKD, and the necessity for long-term RRT (*P* < 0.05), as presented in Table 7.

**Table 7:** Prognostic indicators between the NMF and control group (*x̄* ± *s*) or *M* (*Q*_25_, *Q*__7_5_).

Parameters	The NMF group (*n* = 76)	Control group (*n* = 189)	*t*/*U*/χ^2^	*P*
Cumulative mechanical ventilation duration (*d*)	0.63 (0.50, 0.94)	0.63 (0.50, 0.82)	0.42	0.67
Cumulative ICU stay (*d*)	1.98 (1.70, 3.14)	2.25 (1.75, 2.92)	0.20	0.85
Total hospital stay (*d*)	24.00 (20.00, 31.50)	27.50 (21.00, 36.00)	2.58	0.01
Tracheotomy [*n* (%)]	5 (6.58)	18 (9.52)	0.02	0.89
28-day mortality rate [*n* (%)]	9 (11.84)	16 (8.43)	0.72	0.40
90-day mortality rate [*n* (%)]	10 (13.2)	24 (12.7)	0.19	0.66
RRT-related adverse events [*n* (%)]	7 (9.2)	21 (11.1)	0.24	0.63
CKD [*n* (%)]	11 (14.47)	50 (24.46)	4.39	0.04
CRBSI [*n* (%)]	11 (14.47)	35 (18.52)	0.62	0.43
Requirement for long-term RRT [*n* (%)]	6 (7.89)	35 (18.52)	4.68	0.03
Presence of liver dysfunction [*n* (%)]	42 (55.26)	107 (56.61)	0.04	0.84

ICU, intensive care unit; CKD, chronic kidney disease; CRBSI, catheter-related bloodstream infection; RRT, renal replacement therapy.

##### 3.2.2.4 Propensity score matching (PSM)

We further performed 1:1 nearest-neighbor PSM with a caliper of 0.05. A total of 72 well-matched pairs were successfully generated (144 patients total). Baseline characteristics before and after PSM are summarized in Table 8. Baseline characteristics before and after PSM are summarized in Table 1. Before matching, the NMF group and control group differed significantly in BMI, SOFA score, and APACHE II score (all *P* < 0.05). After 1:1 PSM, 72 well-matched pairs were generated, and all baseline characteristics were well balanced between groups (all *P* > 0.05).

**Table 8:** Baseline characteristics before and after propensity score matching (*x̄* ± *s*) or *M* (*Q*_25_, *Q*_75_).

Characteristic	Before matching			After matching (1:1)		
	NMF group (*n* = 76)	Control group (*n* = 189)	*P*	NMF group (*n* = 72)	Control group (*n* = 72)	*P*
Age, years	54.34 ± 9.35	52.82 ± 8.95	0.22	54.6 ± 9.5	54.0 ± 9.1	0.68
Male, *n* (%)	62 (81.58)	158 (83.60)	0.69	59 (81.9)	59 (81.9)	1.00
BMI, kg/m^2^	23.01 ± 3.28	22.36 ± 2.95	0.12	23.2 ± 3.3	23.2 ± 3.2	0.98
SOFA score	4.00 (3.00, 6.00)	4.00 (3.00, 6.00)	0.80	4.5 ± 2.5	4.7 ± 2.2	0.73
APACHE II score	9.50 (7.00, 14.75)	10.00 (7.00, 13.00)	0.77	10.8 ± 5.2	11.1 ± 3.3	0.71
Operation time, min	526.95 ± 101.19	530.63 ± 106.03	0.80	531.0 ± 103.2	530.2 ± 99.6	0.97
Whole liver transplant, *n* (%)	49 (64.47)	115 (60.85)	0.58	46 (63.9)	48 (66.7)	0.86
Hepatic encephalopathy, *n* (%)	12 (15.79)	25 (13.23)	0.59	11 (15.3)	8 (11.1)	0.62

BMI, body mass index; SOFA, sequential organ failure score; APACHE-II, acute physiology and chronic health II score.

Clinical outcomes are shown in Table 9. In unadjusted analyses, patients in the NMF-guided group had significantly lower postoperative 7-day serum creatinine, shorter total hospital stay, and lower rates of long-term RRT dependence compared with the control group (all *P* < 0.05). After adjustment by multivariate linear regression and binary logistic regression, NMF-guided RRT initiation remained independently associated with: Lower 7-day creatinine (β = −8.01, 95% *CI*: −10.57 to −5.45; *P* < 0.01); Shorter hospital stay (β = −3.38, 95% *CI*: −4.73 to −2.02; *P* < 0.01); Lower risk of CKD (*OR* = 0.44, 95% *CI*: 0.22 to 0.88; *P* = 0.02); Lower risk of long-term RRT dependence (*OR* = 0.20, 95% *CI*: 0.06 to 0.68; *P* = 0.01). No significant differences were observed in 28-day mortality, 90-day mortality, or CRBSI rate (all *P* > 0.05).

**Table 9:** Clinical outcomes in the historically controlled cohort: unadjusted and multivariate-adjusted effects *M* (*Q*_25_, *Q*_75_).

Outcome	NMF group (*n* = 76)	Control group (*n* = 189)	Unadjusted *P*-value	Multivariate adjusted	
				Effect (95% *CI*)	*P*
Postoperative 7-day creatinine, μmol/L	64.45 (50.65, 86.38)	72.20 (61.78, 101.43)	< 0.01	β = −8.01 (−10.57 to −5.45)	< 0.01
Total hospital stay, days	24.00 (20.00, 31.50)	27.50 (21.00, 36.00)	< 0.01	β = −3.38 (−4.73 to −2.02)	< 0.01
CKD, *n* (%)	11 (14.47)	50 (24.46)	0.04	*OR* = 0.44 (0.22 to 0.88)	0.02
Long-term RRT dependence, *n* (%)	6 (7.89)	35 (18.52)	0.03	*OR* = 0.20 (0.06 to 0.68)	0.01
28-day mortality, *n* (%)	9 (11.84)	16 (8.43)	0.40	*OR* = 0.15 (0.02 to 1.23)	0.08
90-day mortality, *n* (%)	10 (13.2)	24 (12.7)	0.66	*OR* = 1.05 (0.46 to 2.38)	0.92
CRBSI, *n* (%)	11 (14.47)	35 (18.52)	0.43	*OR* = 0.70 (0.21 to 2.31)	0.56

CKD, chronic kidney disease; RRT, renal replacement therapy; CRBSI, catheter-related bloodstream infection.

After PSM, the beneficial effects of NMF guidance remained consistent and robust. Patients in the NMF group still exhibited significantly lower 7-day creatinine and shorter hospital stay compared with the control group (both *P* < 0.01; Table 10).

**Table 10:** Clinical outcomes after 1:1 propensity score matching.

Outcome	NMF group (*n* = 72)	Control group (*n* = 72)	*P*
Postoperative 7-day creatinine, μmol/L	63.7 ± 7.9	71.4 ± 9.5	< 0.01
Total hospital stay, days	23.7 ± 4.2	26.6 ± 5.6	< 0.01
CKD, *n* (%)	13 (18.1)	21 (29.2)	0.17
Long-term RRT dependence, *n* (%)	2 (2.8)	7 (9.7)	0.17
28-day mortality, *n* (%)	1 (1.4)	4 (5.6)	0.36
90-day mortality, *n* (%)	10 (13.9)	10 (13.9)	1.00
CRBSI, *n* (%)	4 (5.6)	3 (4.2)	1.00

CKD, chronic kidney disease; CRBSI, catheter-related bloodstream infection.

##### 3.2.2.5 Multivariate regression analysis

Multivariate regression results are presented in Table 11. For continuous outcomes, multivariate linear regression confirmed that NMF-guided RRT was independently associated with significantly lower 7-day serum creatinine and shorter total hospital stay after adjustment for confounding factors. For categorical outcomes, binary logistic regression was performed, and the adjusted results have been summarized in Table 9. No significant independent associations were observed between NMF guidance and 28-day or 90-day mortality.

**Table 11:** Multivariate linear regression for continuous outcomes.

Variable	β-coefficient	95% *CI*	*P*
Postoperative 7-day creatinine			
NMF-guided group (vs. control)	−8.007	−10.566 to −5.448	< 0.01
Age	0.062	−0.185 to 0.309	0.63
BMI	−0.114	−0.582 to 0.354	0.63
SOFA score	0.511	0.065 to 0.957	0.03
APACHE II score	0.155	−0.087 to 0.397	0.21
Total hospital stay			
NMF-guided group (vs. control)	−3.376	−4.729 to −2.023	< 0.01
Age	0.041	−0.063 to 0.145	0.44
BMI	−0.055	−0.254 to 0.144	0.59
SOFA score	0.215	0.023 to 0.407	0.03
APACHE II score	0.088	−0.014 to 0.190	0.09

BMI, body mass index; SOFA, sequential organ failure score; APACHE-II = acute physiology and chronic health II score.

## 4 Discussion

Since 2021, our research team has been engaged in the development of NMF standards. In our initial investigations, we allocated specific weights to NE dose, MELD-Na score, and intraoperative total intake, culminating in the establishment of a straightforward and practical “NMF standards”. Furthermore, we had developed a WeChat mini program designed to more accurately determine the initiation timing of RRT in patients with AKI following liver transplantation, thereby reducing reliance on clinical judgment alone.

Multivariate regression analyses further confirmed the independent protective effects of NMF-guided RRT strategy. After adjusting for major confounding factors, multivariate linear regression showed that NMF guidance was independently associated with lower 7-day serum creatinine and shorter hospital stay. Binary logistic regression demonstrated that NMF-guided RRT independently reduced the risks of CKD and long-term RRT dependence, with adjusted results presented in Table 9.

These findings indicate that the benefits of NMF-based decision-making are not simply caused by baseline differences but represent a genuine clinical advantage of timely and risk—stratified RRT initiation. The consistency of results across univariate analysis, multivariate adjustment, and propensity score matching strengthens the reliability and robustness of the NMF standards in clinical practice.

The MELD-Na score, which incorporates total bilirubin (TBil), ascites-related blood sodium, serum creatinine, and the international normalized ratio (INR), demonstrates superior predictive value for 3-month survival in patients with end-stage liver disease compared to the MELD score alone (17). Additionally, it serves as an independent risk factor for hepatorenal syndrome (18). Recent studies indicate that the implementation of renal RRT can enhance the MELD-Na score in postoperative patients, suggesting effective support and improvement of hepatic and renal function. The timely administration of RRT following liver transplantation, particularly in patients with elevated preoperative MELD-Na scores, has been shown to significantly decrease the incidence of postoperative complications and enhance long-term patient survival rates (19). In a recent study, researchers investigated the prognostic utility of the MELD-Na score in assessing renal function complications following liver transplantation. Evidence indicates that patients whose MELD-Na scores are predominantly influenced by renal dysfunction face a significantly elevated risk of postoperative mortality and renal function complications. This risk is particularly pronounced in older patients, where those with MELD-Na scores comprising 50% or more of the total score experience a 52% increased risk of mortality and an 11-fold higher incidence of severe renal dysfunction 1 year post-surgery (20). The MELD-Na score thus serves as a comprehensive predictor of both survival rates and the likelihood of renal impairment in individuals with end-stage liver disease (21).

The incidence of AKI following liver transplantation is intricately linked to intraoperative fluid management and the administration of NE. Empirical evidence indicates a significant correlation between the accumulation of intraoperative fluid balance and the onset of AKI. Inadequate fluid management during surgical procedures can exacerbate postoperative renal function, particularly in complex operations such as liver transplantation (22). NE is frequently employed to maintain hemodynamic stability during liver transplantation; however, its use may adversely impact postoperative renal function. Studies have demonstrated that patients receiving NE during liver transplantation may experience suboptimal postoperative outcomes. The administration of NE is associated with an increased postoperative heart rate and reduced patient survival, underscoring the need for prudence in its use during liver transplantation (23). Furthermore, intraoperative fluid management and NE administration may also influence renal oxygenation and hemodynamic status post-liver transplantation. Despite the potential for a highly dynamic state in postoperative systemic circulation, renal function may still experience significant decline, primarily due to impaired renal oxygenation. Postoperatively, there is a marked increase in renal oxygen consumption without a corresponding rise in renal oxygen supply, which may be a critical factor contributing to postoperative AKI (24). Extensive blood transfusion and fluid infusion can trigger secondary inflammatory reactions, release oxygen free radicals, and result in renal tubular ischemic necrosis (4) and acute lung injury. Additionally, intraoperative hypotension and the inappropriate use of NE can lead to renal ischemia and hypoperfusion (25). Furthermore, the increase in total intraoperative fluid intake and the administration of NE can elevate both pre- and post-cardiac load, causing a sharp rise in CVP and restricted renal venous return, thereby exacerbating renal dysfunction. Persistent hypotension and extended administration of NE, in conjunction with infection, the use of immunosuppressive agents (notably those with nephrotoxic potential), and the processes of blood transfusion and infusion, may induce renal vascular spasm and capillary endothelial damage. This is often accompanied by the formation of microthrombi, which further diminishes renal perfusion and contributes to the progressive decline of renal function (26).

It is critical to reconcile the present findings with recent high-level evidence regarding RRT timing in critically ill patients. The STARRT-AKI trial (*n* = 2,927) demonstrated that accelerated RRT initiation did not reduce 90-day mortality and was associated with a higher rate of RRT dependence at 90 days compared with the standard strategy (27). An individual patient data meta-analysis by Gaudry et al. (28) further confirmed no significant mortality difference between early and standard RRT strategies and concluded that RRT can be safely delayed in the absence of urgent indications. A 2022 review in the New England Journal of Medicine by Gaudry et al. (29) also emphasized that no biomarkers or clinical scoring systems have been sufficiently validated to guide RRT initiation in the general critically ill population. Notably, these landmark trials were conducted in unselected critically ill patients and may not directly apply to orthotopic liver transplant recipients, who represent a pathophysiologically distinct population. Liver transplant recipients frequently present with high-volume intraoperative fluid overload, high-dose norepinephrine infusion, impaired ammonia metabolism, and elevated preoperative MELD-Na scores—major risk factors for refractory AKI and poor renal recovery. The NMF standard is not a universal early RRT strategy, but a risk-stratified tool to identify high-risk patients who would benefit most from targeted timely RRT. This personalized approach may explain the improved renal outcomes without unnecessary RRT exposure observed in our study.

The research team conducted a retrospective cohort study on 189 liver transplant patients with stage 2 AKI. Results showed that patients meeting NMF standards and receiving RRT had significant improvements in creatinine and blood ammonia levels on postoperative days 1 and 3, and in creatinine, MELD-Na score, and blood ammonia on day 7, compared to those not receiving RRT. Conversely, patients not meeting NMF standards but undergoing RRT only showed improvements in creatinine on day 1 and in creatinine and blood ammonia on day 3, with no significant changes on day 7. This suggests RRT benefits those meeting NMF standards, while benefits are limited for those not meeting the NMF standards.

In historically controlled cohort study, the research team observed that employing the NMF standards to decide the initiation of RRT in patients with AKI stage 2 following liver transplantation resulted in improved clinical indicators, including creatinine levels, MELD-Na scores, blood ammonia, creatinine values on postoperative days 1, and creatinine values on postoperative days 7 compared to decisions based on prior clinical experience. The underlying reasons for these improvements are likely multifactorial. RRT plays a crucial role in reducing blood creatinine levels by eliminating metabolic waste and toxins, which is particularly significant for AKI patients post-liver transplantation. Previous studies have demonstrated that RRT effectively lowers postoperative creatinine levels, thereby enhancing renal function, aligning with the findings of this study. As a therapeutic intervention, RRT not only aids in the enhancement of renal function and reduction of blood creatinine levels but also contributes to improved postoperative survival rates (30). Meanwhile, RRT has demonstrated significant potential in the management of hyperammonemia. Liver failure frequently results in elevated blood ammonia levels, which can precipitate severe neurological complications, including hepatic encephalopathy. Studies have indicated that RRT is efficacious not only in supporting renal function but also in ameliorating neurological symptoms and enhancing overall prognosis by decreasing blood ammonia concentrations. In the context of liver transplantation, the implementation of RRT has been shown to substantially lower blood ammonia levels, thereby positively influencing the reduction of postoperative complications and improving patient survival rates (31, 32).

In terms of prognosis, both retrospective and historically controlled cohort studies suggest that patients who meet the NMF standards and receive RRT generally experience shorter hospital stays and demonstrate a reduced incidence of concurrent CKD and the need for long-term RRT. Conversely, the use of non-targeted RRT increases the risk of bloodstream infections. Evidence indicates that although liver transplant recipients may face a heightened risk of postoperative complications, effective management of RRT can significantly reduce both the duration of hospitalization and the likelihood of persistent renal dysfunction (33). A meticulously planned RRT initiation time not only accelerates postoperative recovery but also has the potential to decrease the occurrence of long-term complications, thus enhancing the overall prognosis for patients.

Limitations: First, the enrollment period spans more than 5 years, and the control group is a historical cohort while the NMF group is a prospective observational cohort. This non-concurrent design introduces potential temporal bias, as advances in perioperative diagnosis and treatment, improvements in surgical techniques, and accumulation of clinical experience among surgeons and ICU physicians over time may have influenced postoperative recovery and renal outcomes. Although baseline characteristics were well balanced between groups, this historical control design is an important methodological limitation, and the findings require further validation in a concurrent controlled trial in the future. Second, several retrospective subgroups were relatively small, including Group A (*n* = 47) and Group C (*n* = 18), which limits statistical power and may reduce the reliability of subgroup comparisons. As an exploratory validation study, the sample size was determined by the number of eligible consecutive patients during the study period. Therefore, the findings should be considered preliminary, and further large-scale studies are required to confirm the reliability and generalizability of the NMF standards.

Third, all comparative analyses in the cohort study were based on unadjusted univariate tests without multivariable adjustment. Although the primary historically controlled cohort was analyzed using multivariate regression and propensity score matching to minimize confounding, residual confounding may still exist. Therefore, the results should be interpreted as associative rather than causal. The derivation process and weighting logic of the NMF standards were initially established in our preliminary research. Although we supplemented the detailed methodology, including ROC curve analysis and clinical rationales for cutoff determination, further refinement and external validation are still needed. Fourth, complete 1-year mortality data were not available for the historically controlled cohort at the time of manuscript preparation, and only 90-day mortality was supplemented and reported in Table 7. Long-term survival data beyond 1 year will be systematically collected and reported in future follow-up studies.

Fifth, this study was conducted in a single Chinese transplant center, with a predominantly male cohort and viral hepatitis as the main etiology of end-stage liver disease, which is highly consistent with the Chinese liver transplant population. Therefore, the generalizability of the conclusions to Western populations with different etiologic profiles, such as NASH and alcoholic liver disease, is limited. Furthermore, only recipients of grafts from donation after brain death (DBD) were enrolled, while donation after cardiac death (DCD) recipients were excluded to ensure population homogeneity. This may also limit the applicability of the findings to liver transplant populations including DCD donors. Finally, due to donor scarcity, there is an increasing reliance on split liver transplantation or live donor liver transplantation. These methods differ from traditional whole liver transplantation and may affect postoperative outcomes. This study is limited by the single-center design and relatively small sample size, necessitating further prospective research to confirm our findings.

In conclusion, our research team has successfully developed the NMF standards and implemented it through the creation of the “NMF standards” WeChat mini program. Concurrently, we had secured the computer software copyright for “NMF Standard Risk Assessment Software V1.0.” Retrospective and historically controlled cohort studies conducted by our team indicated that patients undergoing RRT in accordance with the NMF standards exhibit significant improvements in postoperative creatinine levels, MELD Na scores, and blood ammonia levels, in addition to experiencing reduced overall hospital stays. Furthermore, the incidence of concurrent CKD and the necessity for long-term RRT were notably lower among these patients. The NMF standards may demonstrate substantial clinical applicability in determining the optimal timing for RRT in patients with AKI following liver transplantation.

## Data Availability

The datasets presented in this study can be found in online repositories. The names of the repository/repositories and accession number(s) can be found below: Dong, Zhouzhou (2025), “Timing of Renal Replacement Therapy in Acute Kidney Injury After Liver Transplantation: The Value of NMF Standards”, Mendeley Data, V1, doi: 10.17632/vhncc43xwx.1.
